# Pan-Genome-Wide Analysis and Expression Profiling of the Potato GST Gene Family

**DOI:** 10.3390/plants15101548

**Published:** 2026-05-19

**Authors:** Ming Li, Jinyong Zhu, Zhitao Li, Xiaoqiang Qiu, Minmin Bao, Zhijie Chen, Zhenzhen Bi, Chao Sun, Yuanming Li, Zhen Liu, Yuhui Liu

**Affiliations:** 1 State Key Laboratory of Aridland Crop Science (Gansu Agricultural University), Gansu Agricultural University, Lanzhou 730070, China; 1073324120385@st.gsau.edu.cn (M.L.); zhujinyong@gsau.edu.cn (J.Z.); 1073324020175@st.gsau.edu.cn (Z.C.); 2 College of Agronomy, Gansu Agricultural University, Lanzhou 730070, China; lizt@st.gsau.edu.cn (Z.L.); 1073323020267@st.gsau.edu.cn (X.Q.); 1073323020285@st.gsau.edu.cn (M.B.); bizz@gsau.edu.cn (Z.B.); sunc@gsau.edu.cn (C.S.); 3 College of Horticulture, Gansu Agricultural University, Lanzhou 730070, China; liyuanm@gsau.edu.cn

**Keywords:** pan-gene family, auxin, differential expression, abiotic stress, potato

## Abstract

Glutathione S-transferases (GSTs) are an important family of enzymes involved in plant detoxification, maintenance of redox homeostasis, and responses to abiotic stresses. However, the evolutionary characteristics and functional roles of the potato GST pan-gene family have not yet been systematically investigated at the pan-genome level. In this study, based on high-quality potato genomes constructed from 45 diploid accessions, GST gene family members were systematically identified, and their evolutionary features and expression patterns were analyzed. Phylogenetic analysis classified the GST family into six subgroups, among which the soft-core gene *StGST7* and the near-core genes *StGST8* and *StGST16* were assigned to the Phi and Tau subgroups, respectively. Selection pressure analysis indicated that five *StGST* genes may have undergone positive selection, whereas most of the remaining genes were mainly subjected to purifying selection. Structural variation significantly affected the expression of *StGST42* and the conserved domains of its encoded protein. Expression profiling revealed that GST family members exhibited clear tissue-specific expression patterns and responded differentially to drought, salt, high temperature, ABA, and IAA treatments. Co-expression network analysis revealed significant positive and negative correlations between multiple transcription factors and *StGST* gene expression, suggesting their potential involvement in the coordinated regulation of *StGST* genes. Further analyses demonstrated that *StGST7* was significantly differentially expressed under multiple stress conditions, and its heterologous expression enhanced yeast tolerance to salt and drought stress. This study revealed the evolutionary characteristics and potential functions of the potato GST gene family and provides a theoretical basis for elucidating the molecular mechanisms underlying its regulation of environmental adaptation.

## 1. Introduction

Glutathione S-transferases (GSTs) constitute a large and ubiquitous superfamily of multifunctional enzymes that are widely distributed across both eukaryotic and prokaryotic organisms. In plants, GSTs are encoded by multigene families and are broadly expressed throughout different stages of growth and development [1,2]. The primary biochemical function of GSTs is to catalyze the conjugation of reduced glutathione (GSH) to a wide range of endogenous and exogenous toxic compounds [3], thereby facilitating their detoxification, sequestration, and removal. Through these processes, GSTs play a pivotal role in cellular detoxification, redox homeostasis, and stress adaptation [4,5,6]. Structurally, a typical GST protein consists of an N-terminal domain responsible for GSH binding and a C-terminal domain involved in the recognition of hydrophobic substrates [7]. Based on conserved structural features, plant GSTs are classified into 14 subclasses, including phi (F), tau (U), zeta (Z), theta (T), lambda (L), dehydroascorbate reductase (DHAR), tetrachlorohydroquinone dehalogenase (TCHQD), glutathione–hydroquinone reductase (GHR), elongation factor 1Bγ (EF1Bγ), hemerythrin (H), iota (I), ure2p, metaxin, and microsomal prostaglandin E synthase 2 (mPGES-2) [8]. Notably, glutathione S-transferase (GST) subfamilies such as Tau, Phi, Lambda, and TCHQD constitute a highly versatile biochemical defense network in plants. They not only efficiently conjugate with harmful xenobiotic compounds, such as insecticides and herbicides, to mitigate cytotoxicity, but also actively participate in critical physiological processes including reactive oxygen species (ROS) scavenging under abiotic stress, regulation of secondary metabolism, and hormone signaling. Relying on this multi-tiered mechanism of detoxification and homeostasis maintenance, these GST subfamilies comprehensively enhance plant adaptability to multiple environmental stresses [9,10]. Consequently, this molecular family occupies an extremely vital position in plants. Although earlier views considered them to be exclusively plant-specific [11], subsequent research—such as the discovery of the Phi class widely distributed in non-plant taxa [12]—has demonstrated that this perspective is not entirely accurate. Under adverse environmental conditions, excessive accumulation of reactive oxygen species (ROS) frequently occurs in plants, leading to oxidative stress and damage to cellular components, including lipids, proteins, carbohydrates, and nucleic acids [13]. To counteract ROS-induced damage, plants have evolved sophisticated antioxidant defense systems. GSTs, as central components of plant antioxidant defense and stress adaptation systems, are not only involved in regulating ROS signaling, redox balance, and hormone biosynthesis, but also play a key role in responses to various abiotic stresses, including salinity, drought, and extreme temperatures [14]. To date, a large family of *GST* genes has been widely identified in a variety of plants, including *Arabidopsis thaliana* (61 genes) [15], *Brassica oleracea* (179 genes) [16], and *Hordeum vulgare* (84 genes) [8,16,17,18,19]. At the functional validation level, a growing body of research consistently demonstrates that the activation of specific GST members can significantly enhance plant stress tolerance through diverse mechanisms [4]. Regarding ROS scavenging and stress tolerance, *SlGSTU43* in tomato (*Solanum lycopersicum*) confers salt resistance by efficiently scavenging excessively accumulated ROS and promoting lignin biosynthesis [20], whereas *PtGSTF1* in poplar (*Populus tremula*) acts protectively primarily by improving ion homeostasis under salt stress [21]. In terms of cold tolerance regulation, the overexpression of the *JrGSTU1* gene in walnut (*Juglans regia*) significantly bolsters the plant’s resistance to cold stress [22], and *IbGST4* and *IbGST2* in sweet potato (*Ipomoea batatas*) also maintain cell viability by effectively alleviating cold-induced oxidative damage [23]. For multiple stresses, *VvGSTF13* in grapevine (*Vitis vinifera*) [24] and *ThGSTZ1* in *Tamarix hispida* [25,26] demonstrate dual efficacies in enhancing antioxidant capacity and drought/salt tolerance. Additionally, in rice (*Oryza sativa*), the overexpression of *OsGSTU4* has been proven to significantly reduce stress-induced ROS accumulation, thereby improving drought and salinity tolerance [5].

Beyond their enzymatic roles in antioxidant detoxification, GSTs also act as non-enzymatic carriers (ligand-binding proteins) deeply involved in the vacuolar transport of secondary metabolites [27,28]. For instance, in *Arabidopsis thaliana*, while *AtGSTU17* regulates seed development and the accumulation of secondary metabolites [29], the classic *AtGSTF12* (also known as *TT19*) has been proven responsible for the safe transport of anthocyanins and proanthocyanidins from the cytoplasm into the vacuole for sequestration. In recent years, specific GST members in horticultural crops, such as apple (*Malus domestica*) [30] and strawberry (*Fragaria ananassa*) [28], have also been identified as key transporters for antioxidant flavonoids like anthocyanins. This targeted transport not only determines the coloration of plant organs but also provides a crucial biochemical barrier against external stresses such as UV radiation, further highlighting the multifunctionality of the GST family in plant stress adaptation.

*GST* genes not only exhibit a high degree of versatility in their biochemical functions, but their transcriptional activation is also governed by complex and precise upstream regulatory networks. For instance, when tea plants (*Camellia sinensis*) are subjected to drought stress, the transcription factor CsWRKY48 can specifically bind to and upregulate the expression of *CsGSTU8*, thereby conferring drought tolerance [31]. Moreover, GSTs display remarkable sensitivity and a broad-spectrum response to multiple environmental stimuli. For example, heavy metal cadmium treatment significantly activates GST enzyme activity in pea (*Pisum sativum*) to combat toxic damage [32]; additionally, the heterologous expression of the rice (*Oryza sativa*) *OsGSTU4* and *OsGSTU30* genes in *Arabidopsis* enhances the comprehensive tolerance of transgenic plants to various combined stresses, including drought and heavy metals [5,33].

Taken together, whether through antioxidant detoxification, secondary metabolite transport, or responses to complex transcriptional regulatory signals, the massive GST gene family acts as an irreplaceable hub in plant defense against adverse environments. However, precisely due to the massive expansion of GST members, their sequence diversity, and the fact that their functions are often modulated by the cross-talk of multiple stress factors, comprehensively decoding this network and effectively utilizing key *GST* genes for the targeted breeding of highly resilient crops remains a major challenge in plant molecular breeding. This necessitates more systematic and in-depth genomic and functional investigations.

Potato (*Solanum tuberosum*) is an annual herbaceous crop belonging to the genus Solanum within the family Solanaceae [34]. Originating in the Andean region of South America [35], potato is currently the world’s third most important food crop after rice and wheat (*Triticum aestivum*) [36,37,38]. Owing to its high nutritional value, strong adaptability, and stable yield, the potato plays a crucial role in global food security. However, potato production is severely constrained by abiotic stresses, including drought, high temperatures, and salinity. Starch metabolism, which governs carbohydrate allocation to tubers and directly influences tuber yield and quality, represents a central physiological process affected by environmental stress [39]. In this context, comprehensive gene family analyses provide powerful approaches for systematically elucidating the genetic basis of stress responses and agronomic traits, and such approaches have been successfully applied in crops including rice [40], and barley [41].

To better understand the evolutionary dynamics and functional divergence of GSTs in *Solanum tuberosum*, this study aimed to comprehensively characterize the *StGST* gene family at the pan-genome level. By integrating structural, phylogenetic, and expression profiling analyses under various stress conditions, we sought to identify key candidate genes responding to environmental stimuli, thereby laying a solid foundation for future stress-resilience breeding in potato.

## 2. Results

### 2.1. Identification of the Potato Pan-Genome and Analysis of Presence/Absence Variations

**Figure 1:**
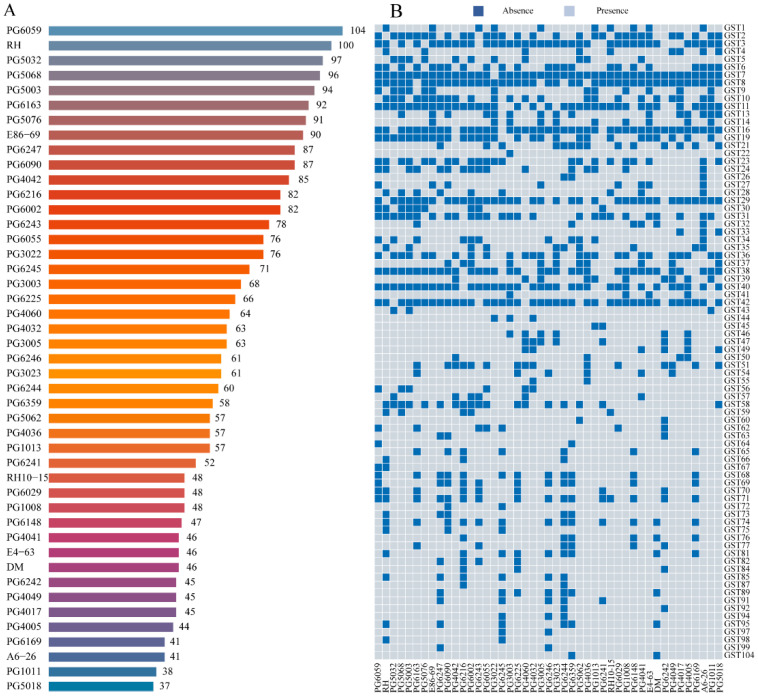
Presence/absence variation (PAV) analysis of the *StGST* gene family in the potato pan-genome. (**A**) Statistical summary of the number of *StGST* family members identified across different potato lines. (**B**) The PAV landscape of *StGST* members among the analyzed potato genomes. To ensure the robustness of the analysis, only gene members present in at least two potato lines were retained for further evaluation. In the heatmap, dark blue grids indicate the presence of the gene in a specific line, while light blue grids indicate its absence.

To systematically analyze the genome-wide characteristics of the potato glutathione S-transferase (GST) gene family, this study identified 2940 GST gene family members across 45 diploid potato lines through systematic analysis of the potato pan-genome (Table S1). Analysis revealed significant variation in the number of *GST* genes among potato varieties, with the PG6059 variety having the highest number (104 genes) and the PG5018 variety the lowest (37 genes) (Figure 1A). Based on gene distribution frequency across the pan-genome accessions, *GST* genes were categorized into distinct types: *StGST7* was classified as a soft-core gene (≥95%), while *StGST8* and *StGST16* were classified as near-core genes (90–95%). 78 *StGST* genes were designated as non-essential genes (5–90%), and the remaining 2859 *StGST* genes, which were detected in only a single accession, were classified as accession-specific genes. Further analysis revealed that certain *StGST* genes exhibit highly specific distribution patterns, co-occurring only in two distinct accessions. Examples include *StGST104* (PG6359 and DM), *StGST45* (PG6241 and PG1013), and *StGST55* (PG4032 and PG4036) (Figure 1B).

### 2.2. Phylogenetic Analysis of Potato GST Genes

**Figure 2:**
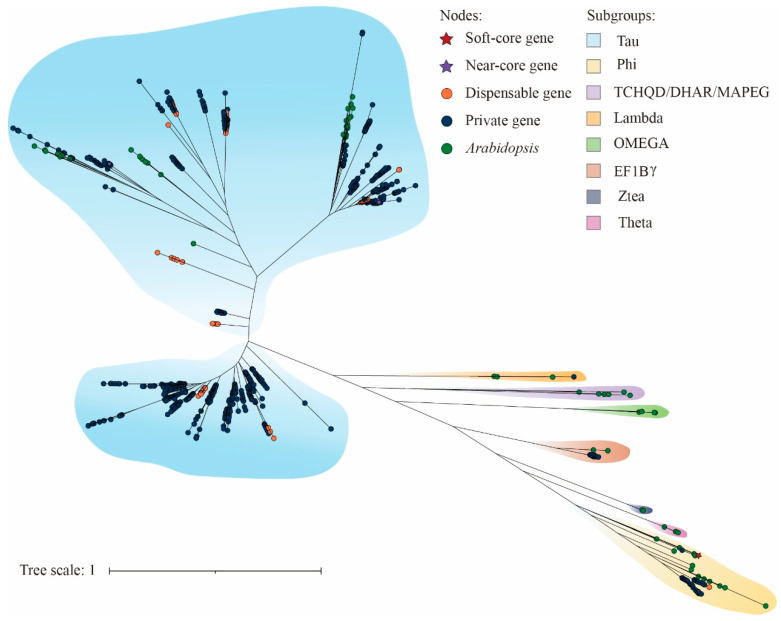
Phylogenetic tree of GSTs from *Arabidopsis* and potato. Node shapes and colors indicate different gene categories: red stars represent soft-core genes, purple stars represent near-core genes, orange circles represent dispensable genes, dark blue circles represent private genes, and green circles represent *Arabidopsis* genes. Different background colors indicate the subgroups to which the genes belong, including Tau, Phi, TCHQD/DHAR/MAPEG, Lambda, OMEGA, EF1Bγ, Zeta, and Theta. Branch lengths represent genetic distances among sequences, with a tree scale of 1.

To clarify the evolutionary relationships and classification characteristics of the potato GST gene family, a maximum-likelihood phylogenetic tree was constructed using 1695 potato GST protein sequences (StGSTs) and 61 *Arabidopsis* GST protein sequences (AtGSTs), with 1000 bootstrap replicates used to evaluate the reliability of the tree. According to the subgroup classification criteria established for the Arabidopsis GST gene family, GST proteins were classified into ten subfamilies: Phi, TCHQD, Theta, Omega, Zeta, MAPEG, DHAR, EF1Bγ, Lambda, and Tau (Figure 2). However, StGSTs were assigned to only six of these subgroups. Among them, the Tau subfamily was the largest, comprising 1529 members, followed by the Phi, EF1Bγ, and Zeta subfamilies, with 108, 38, and 10 members, respectively. The Omega and Lambda subfamilies each contained five members. In contrast, no *StGST* members were identified in the Theta, TCHQD, DHAR, and MAPEG subgroups. Notably, the soft-core gene *StGST7* and the near-core genes *StGST8* and *StGST16* were assigned to the Phi and Tau subgroups, respectively, suggesting that these genes may play important roles in maintaining evolutionary conservation and core functions within the potato GST gene family.

### 2.3. The GST Gene in Potatoes Is Subject to Differential Selection Pressure Across Different Varieties

**Figure 3:**
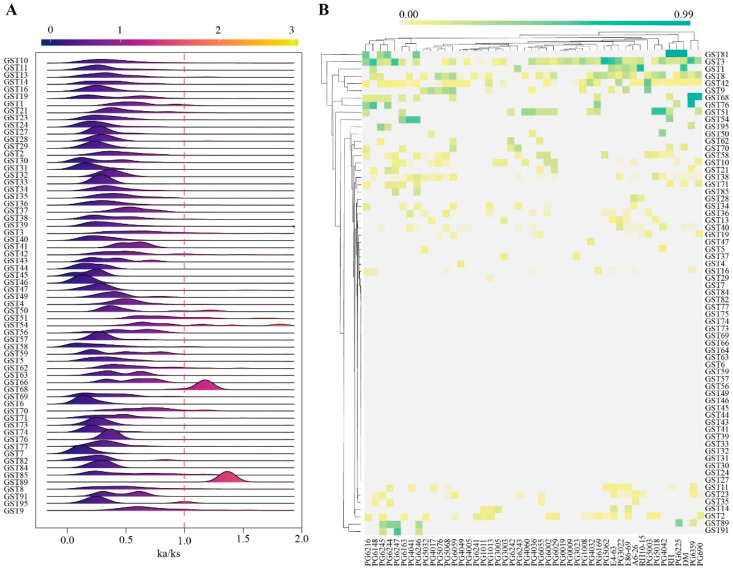
Ka/Ks ratio values for GSTs. (**A**) Ka/Ks values of GSTs in the potato pan-genome; (**B**) heatmap of the frequency of GSTs family members with Ka/Ks > 1 in the genome (gray: the Ka/Ks ratio of this gene in this line was less than 1).

Analysis of the Ka/Ks ratio provides crucial evidence for elucidating the selective pressures experienced by gene family members during the evolution of different varieties. To investigate the selective pressures exerted on potato *StGST* genes during domestication and varietal differentiation, this study calculated the ratio of non-synonymous to synonymous substitution rates (Ka/Ks) for each *StGST* gene using pan-genomic data from 45 representative varieties. As depicted in Figure 3A, the Ka/Ks values for the vast majority of *StGST* genes clustered between 0 and 1, indicating that they were primarily constrained by purifying selection during evolution, with relatively conserved functions. Notably, *StGST68* and *StGST89* exhibited peak Ka/Ks values of 1–2 across multiple accession, suggesting these genes may have undergone adaptive evolution through positive selection in certain genetic backgrounds. Furthermore, a significant proportion of Ka/Ks ratios for *StGST3*, *StGST68*, *StGST76*, *StGST81*, and *StGST89* exceeded 1 (Figure 3B), indicating that these genes were under selective pressure during potato domestication. In contrast, most other genes exhibited Ka/Ks ratios greater than 0 but less than 1, suggesting they were predominantly subject to purifying selection throughout domestication.

### 2.4. The Expression and Structure of the StGST42 Gene Are Affected by Structural Variations (SVs)

**Figure 4:**
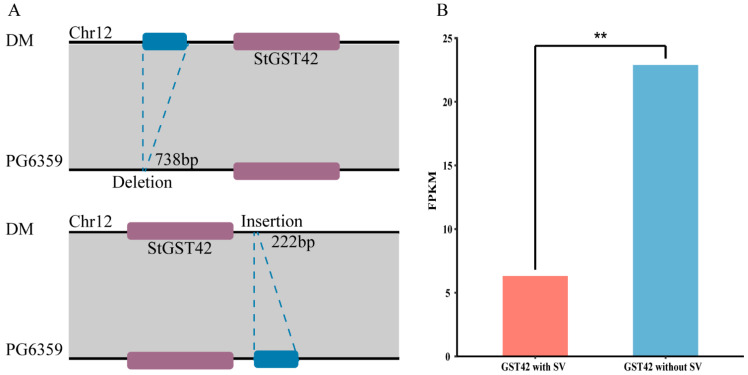
The impact of structural variations on genes. (**A**) The effects of insertional and deletional structural variations on *StGST42*. Compared to DM, the *StGST42* gene on chromosome 12 of PG6359 has a 738 bp deletion in the upstream region and a 222 bp insertion in the downstream region. (**B**) Structural variations significantly affected the expression level of *StGST42*. The expression levels of *StGST42* were compared between potato accessions with and without the deletion using the Wilcoxon rank-sum test. ** *p* < 0.01 indicates a highly significant difference.

To elucidate the impact of structural variations (SVs) on *StGST* gene expression, this study identified 736 SVs within the coding regions and flanking 2 kb regions of 62 *StGST* genes. Compared to the reference genome, these variations primarily manifested as deletions, insertions, and inversions (Figure 4A). By correlating gene expression levels between samples with and without SVs, we found that only *StGST42* expression was significantly affected by SVs (Pearson correlation coefficient |r| > 0.3, *p* < 0.01) (Figure 4B). These results suggested that SVs may specifically regulate their expression by altering the genomic environment surrounding this gene. Notably, varieties DM and PG6359 exhibit significant genetic divergence at the *StGST42* locus, potentially reflecting their distinct geographical origins.

**Figure 5:**
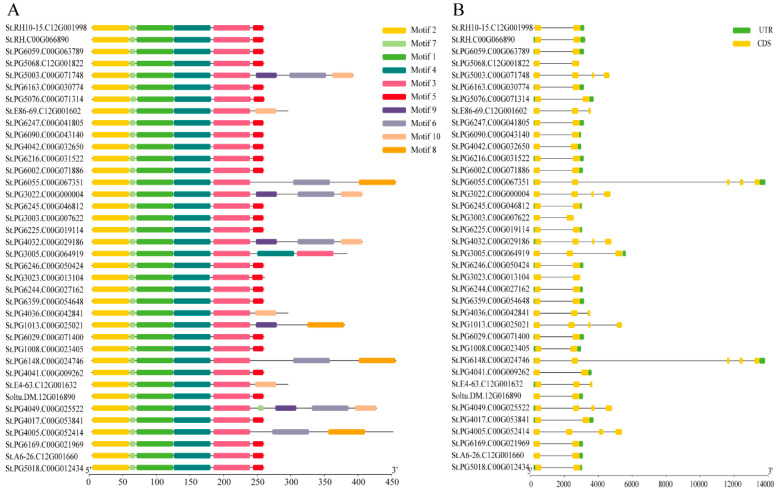
Conserved motif composition and gene structure analysis of the StGST gene family. (**A**) Distribution patterns of conserved motifs in *StGST* genes. Ten highly conserved motifs were identified using the MEME Suite (represented by differently colored boxes), highlighting their structural conservation across various potato accession. (**B**) Gene structures of *StGST* genes across 39 potato lines. The diagram visually details the relative positions and lengths of exons, introns, and untranslated regions (UTRs) within each gene.

The conserved structure of the StGSTs was analyzed using the online program MEME, and a total of 10 conserved motifs were identified, which were sequentially named motif 1 to motif 10 (Figure 5A). Among these, motif 2, motif 7, motif 1, motif 4, and motif 3 were present in all 39 StGSTs, indicating these motifs are highly conserved within the family. Motif 5 is similarly widely distributed across the majority of members, absent only in 13 proteins, including PG5003 and E8669. Furthermore, motifs 9, 6, motif 10, and motif 8 are present in 5, 8, 7, and 5 StGSTs, respectively. Notably, St.PG5003, St.PG3022, St.PG4032, and St.PG4049 simultaneously contain motifs 9, 6, and 10. These findings indicate that protein sequences within the StGST family exhibit relatively high levels of conservation.

To investigate the impact of insertion and deletion on the gene structure of the GST gene family, we analyzed the structure of the StGST genes in 39 samples using TBtools (v2.420). Results indicate that across most potato varieties, the domain types and numbers, exon composition, and 5′ untranslated regions (UTRs) of StGSTs align with those of the reference genome (DM) (Figure 5B). However, variations in the number of units within the 3′ untranslated region were observed in certain varieties, including PG5068, PG5003, E86-69, PG6055, PG3022, PG3003, PG4032, PG3002, PG4036, PG1013, PG6148, E4-63, PG4009, and PG4005. Notably, PG6055 and PG6148 possess three additional units in their 3′ untranslated regions compared to DM. The remaining genes also exhibit varying degrees of structural variation, which may further alter their functional properties.

### 2.5. Co-Linearity Analysis of GST Genes in Potatoes and Other Plants

**Figure 6:**
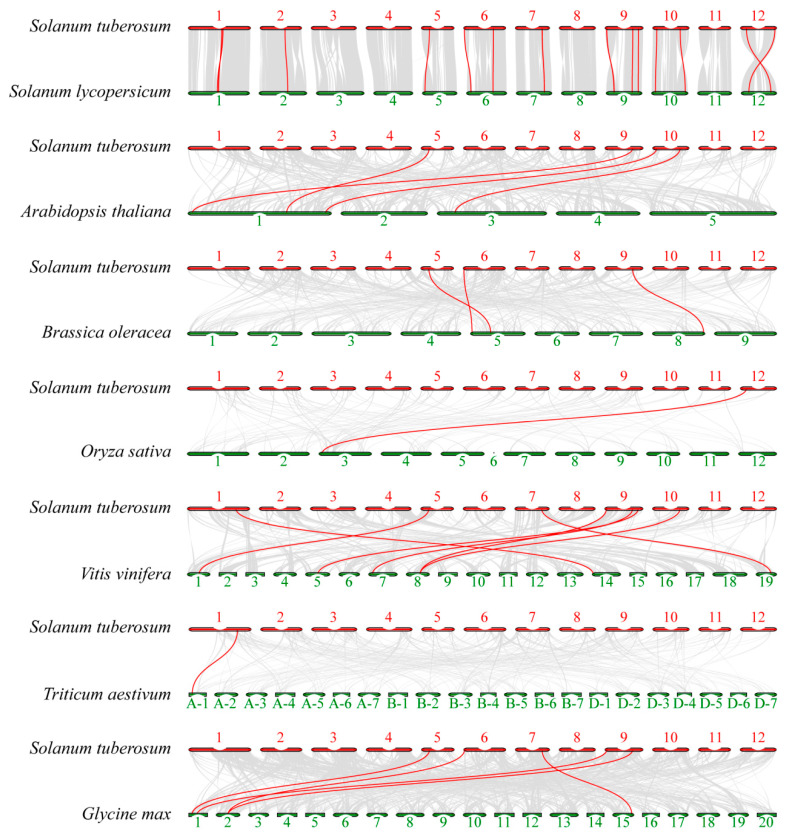
Synteny analysis of *GST* genes between potato and other representative plant species. The comparative genomic analysis illustrates the collinear relationships between potato and diverse plant species, including tomato, *Arabidopsis thaliana*, *Brassica oleracea*, rice, grapevine, wheat, and soybean. The gray lines in the background represent the genome-wide collinear blocks, while the highlighted red lines specifically trace the collinear *GST* orthologous gene pairs.

To clarify the evolutionary trajectory of the glutathione S-transferase (GST) gene in potatoes and their related species, this study conducted a systematic analysis of the genomic structural homology and differentiation patterns between potatoes and their close relatives in the Solanaceae family (tomato), as well as distant relatives in the Brassicaceae (*Arabidopsis thaliana*, *Brassica oleracea*), Gramineae (rice, wheat), Fabaceae (*Glycine max*), and Vitaceae (grape) families (Figure 6). Whole-genome synteny analysis revealed highly corresponding chromosomal synteny between potato and tomato, reflecting significant genomic structural conservation within the Solanaceae. In contrast, a multi-to-one, fragmented synteny pattern emerged with Brassicaceae species such as *Arabidopsis* and *Brassica oleracea*, suggesting independent genomic duplication and rearrangement events following family-level divergence. Conversely, homologous signals were markedly diminished and sparsely distributed among monocotyledons such as rice and wheat, reflecting profound genomic structural divergences accumulated during the extended evolutionary history separating monocotyledons and dicotyledons. At the GST gene family level, 25 pairs of homologous *GST* genes were identified between potato and tomato, further corroborating their close phylogenetic relationship. Four, three, one, seven, one, and five pairs of homologous genes were detected in *Arabidopsis*, *Brassica oleracea*, rice, grape, wheat, and soybean, respectively. Notably, these distantly homologous genes exhibit some spatial conservation in chromosomal position. For instance, genes corresponding to *Arabidopsis* and *Brassica oleracea* are predominantly located on potato chromosomes St-5 and St-9, while those corresponding to rice and wheat are concentrated on chromosome St-12. These results indicated that even during distant evolutionary processes, the GST gene family retains a micro-collinearity structure within local regions.

### 2.6. Analysis of GST Gene Family Expression in Different Tissues of DM Potato Under Abiotic Stress Conditions

**Figure 7:**
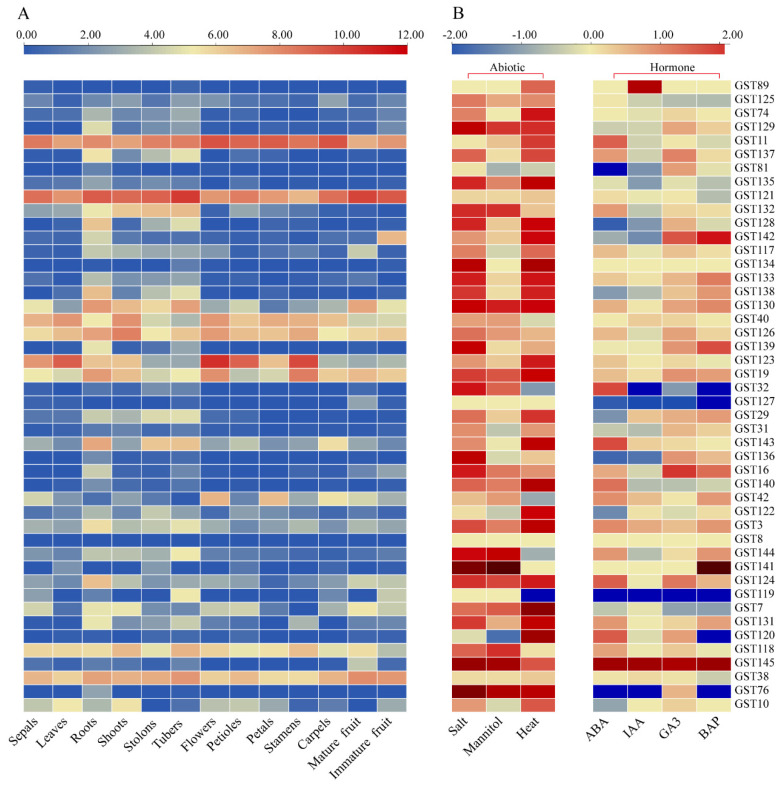
Expression pattern analysis of the potato *StGST* gene family. (**A**) Expression profile of the *StGSTs* in 13 different tissues of the DM potato (sepals, leaves, roots, shoots, stolons, tubers, flowers, petioles, petals, stamens, carpels, mature fruit, and immature fruit). The color in the heat map represents the FPKM value using a logarithm with base 2 (log_2_FPKM). (**B**) Expression responses of *StGSTs* under abiotic stresses (including salt, mannitol, and high temperature stress) and exogenous hormone (ABA, IAA, GA3, and BAP) treatments. The color represents the fold change using a base-2 logarithm (log_2_FC).

Gene expression levels in potato organs at different developmental stages are closely correlated with their biological functions. To construct a comprehensive gene expression profile for the systematic analysis of the functional diversification of the *StGST* gene family, we performed quantitative analysis of RNA-seq data from 13 different tissues, including sepals, leaves, roots, shoots, stolons, tubers, flowers, petioles, petals, stamens, pods, and mature and immature fruits (Figure 7A; see Table S2 for raw quantification results for each organ). Clustering analysis revealed distinct expression specificity of *StGST* genes across tissues. For instance, *StGST11*, *StGST121*, *StGST130*, *StGST132*, *StGST126*, *StGST143*, *StGST118*, and *StGST38* exhibited significantly higher expression in tubers; *StGST40* and *StGST123* exhibited higher expression in leaves; *StGST19* showed preferential expression in roots; and *StGST11*, *StGST121*, *StGST143*, and *StGST38* were also markedly upregulated in stolons. Furthermore, *StGST89*, *StGST134*, *StGST127*, and *StGST136* exhibited low expression levels across most tissues, displaying a constitutive expression pattern. These expression characteristics suggest that members of this gene family may participate in tissue-specific physiological processes. Specifically, gene clusters highly expressed in tubers (e.g., *StGST11*, *StGST121*) may be associated with storage substance accumulation, secondary metabolism, or stress adaptation. In contrast, genes highly expressed in leaves (e.g., *StGST123*) may be more involved in reactive oxygen species scavenging or photoprotection mechanisms associated with photosynthesis.

To further elucidate the role of the GST gene family in stress responses, we systematically analysed its expression profiles under abiotic and biotic stress conditions, as well as in response to exogenous hormones (including salt, mannitol, high temperature, IAA, ABA, GA3 and BAP) (Figure 7B; see Table S3 for detailed RNA-seq quantification data). Results revealed that numerous *StGST* genes exhibited significant transcriptional regulatory responses to different stresses. Under abiotic stresses, salt, mannitol, and high temperature induced differential expression (|log_22_FC| > 1) in 29, 16, and 28 *StGST* genes, respectively. Some genes exhibited cross-stress responsiveness. For instance, *StGST89* and *StGST117* were significantly upregulated under both salt and high-temperature stress, while all three abiotic stresses induced *StGST74* and *StGST129*. Among hormone treatments, abscisic acid (ABA) significantly upregulated *StGST32*, *StGST143*, and *StGST124* expression; indoleacetic acid (IAA) specifically induced *StGST89* and *StGST145*; and gibberellic acid (GA3) promoted *StGST16*, *StGST124*, and *StGST145* expression. Notably, *StGST124*, *StGST142*, and *StGST145* exhibited sustained upregulation responses to ABA, IAA, and GA3, suggesting they may exert broad regulatory functions within hormonal signaling networks.

### 2.7. Expression of Tetraploid Potato StGSTs Under Salt Stress Conditions

**Figure 8:**
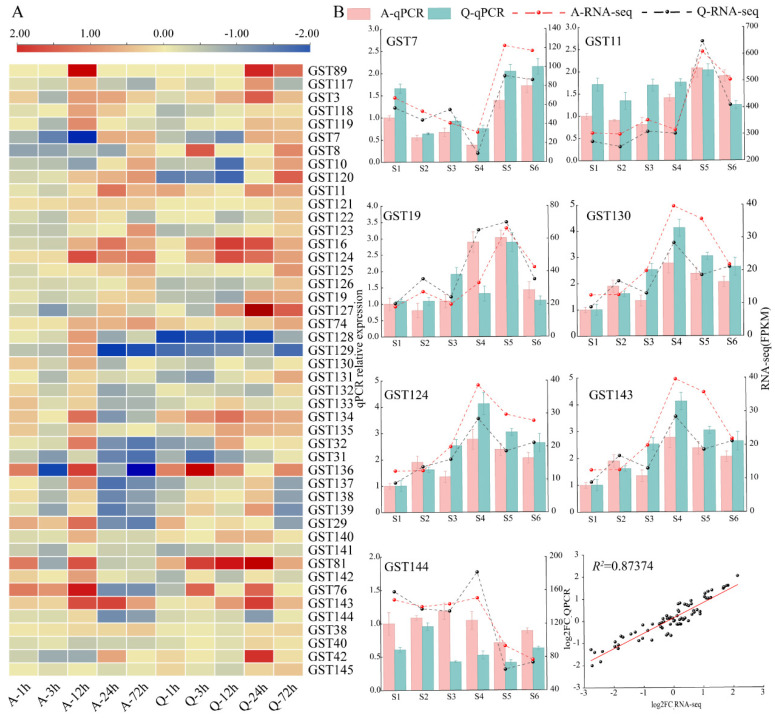
Expression profiles of *StGST* genes in two potato varieties (‘Atlantic’, A; ‘Qingshu No. 9’, Q) under salt stress. (**A**) Time-course transcriptomic expression heatmap of *StGST* genes under salt stress in cultivars A and Q. The color scale represents the log_2_FC value of each gene. (**B**) The qPCR expression analysis of seven candidate *StGST* genes in cultivars A and Q under salt stress at different time points: 0 h (S1), 1 h (S2), 3 h (S3), 12 h (S4), 24 h (S5), and 72 h (S6). The relative expression levels were normalized to the A variety at 0 h (S1), which was set to 1. Data are presented as the mean ± standard error (SE) of three biological replicates, with error bars indicated above the columns.

Analysis of the expression patterns of *StGST* genes under salt stress revealed that the expression of most family members underwent significant changes following stress; not only did they exhibit distinct temporal dynamics, but significant inter-varietal differences were also observed between the sensitive variety (Atlantic) and the drought-tolerant variety (Qingshu No. 9) (Figure 8A; see Table S4 for detailed expression data between the two varieties). During the early stress phase (1–12 h), several *StGSTs* (e.g., *StGST76*, *StGST143*) were rapidly upregulated, suggesting their potential role in early salt stress responses. In contrast, other *StGSTs* (e.g., *StGST89*, *StGST11*) showed sustained increases during the late phase (24–72 h), indicating possible involvement in long-term acclimation. Furthermore, some *StGSTs* (e.g., *StGST7*, *StGST10*) displayed a biphasic pattern, characterized by initial downregulation followed by upregulation over the stress period. Inter-varietal comparisons revealed that while some genes displayed similar expression trends in both varieties, ‘Atlantic’ exhibited earlier responses and larger expression amplitudes for specific *StGSTs*, whereas related genes in ‘Qingshu No. 9’ showed more moderate expression fluctuations. These results suggested that the two varieties may employ distinct salt tolerance strategies at the transcriptional regulation level. The differentially expressed *GST* genes identified may be involved in processes such as reactive oxygen species (ROS) scavenging and detoxification of toxic or secondary metabolites, providing crucial insights for elucidating the molecular mechanisms underlying potato salt stress responses.

To validate the reliability of the RNA-seq dataset, we selected seven *StGSTs* with high and/or differential expression in ‘Qingshu No. 9’ and verified them via qPCR. The results confirmed that the qPCR expression patterns were consistent with the RNA-seq dataset (Figure 8B). The RNA-seq datasets from both potato varieties under salt stress exhibited a strong linear relationship with qPCR results (*y* = 0.6908*x* + 0.1458; *R*^2^ = 0.87347), indicating excellent concordance between the two analytical methods.

### 2.8. Co-Expression Network Analysis of the StGST Gene Family

**Figure 9:**
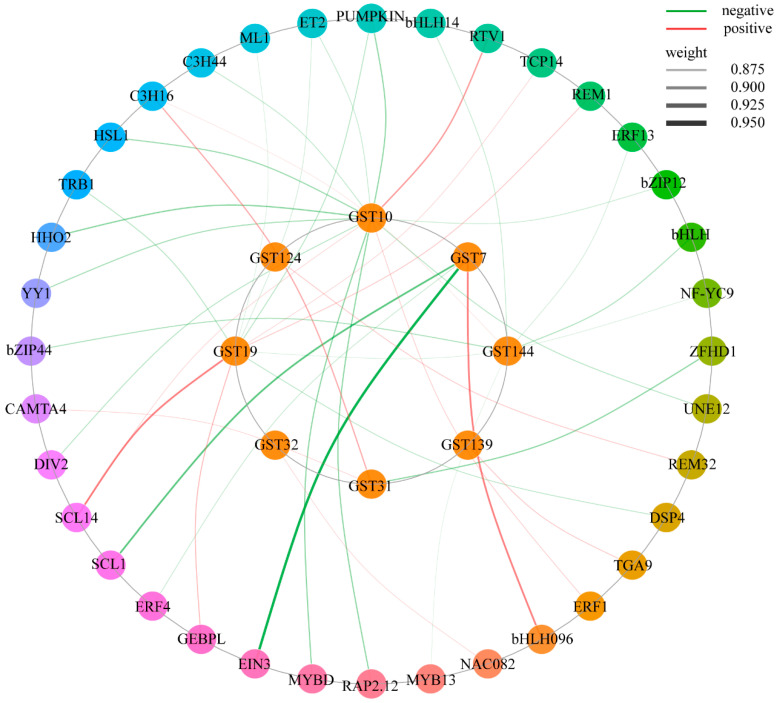
Co-expression network analysis of *StGST* genes and transcription factor families. The network visualizes the expression correlations between the core *StGST* members (central nodes) and other interacting genes (peripheral nodes, such as various transcription factors). The different sizes of the circles represent specific gene attributes (e.g., expression levels or node degrees). The lines connecting two nodes indicate their co-expression relationships: red lines represent significant positive correlations, while green lines represent significant negative correlations. The thickness of the lines intuitively reflects the strength of the correlation between the nodes.

Co-expression network analysis provides a powerful tool for deciphering regulatory relationships between transcription factors and target genes. This study constructed a regulatory network linking glutathione S-transferase (GST) family genes and their upstream transcription factors based on high-confidence weighted co-expression correlation coefficients (0.875–0.950), aiming to reveal the intricate regulatory patterns of transcription factors governing this core metabolic gene family. Transcription factors act as “molecular switches” that specifically regulate gene transcription. By precisely activating or repressing the expression of downstream target genes, they coordinate complex physiological responses during plant growth, development, and environmental stress. As shown in Figure 9, the co-expression network analysis indicated that, under salt stress conditions, members of the *StGST* gene family exhibited significant expression correlations with multiple transcription factor (TF) families, including AP2/ERF, MYB, bZIP, and bHLH. Different *StGST* genes displayed distinct co-expression patterns with specific combinations of TFs, suggesting their association with distinct expression contexts during the salt stress response. For example, *StGST19* showed positive correlations with SCL14 from the SCL family and GEBPL from the bZIP family, while exhibiting a negative correlation with TRB1 from the SANT/MYB family. In addition, *StGST10* displayed complex multidirectional expression correlations with YY1 (GLI-Krüppel family), HSL1 (GRAS family), RTV1 (Retromer complex), and HHO2 (GARP family). Under salt stress, *StGST7* exhibited expression patterns distinct from those of other members. Its expression level increased progressively with prolonged treatment duration, and it was more highly expressed in the salt-tolerant cultivar ‘Qingshu No. 9’ than in the salt-sensitive cultivar ‘Atlantic’, suggesting a possible association between its expression dynamics and salt tolerance. Further co-expression analysis showed that *StGST7* was positively correlated with bHLH096, while negatively correlated with EIN3 and SCL4. Whether these TFs directly or indirectly regulate the expression of *StGST* genes and thereby participate in potato’s response to salt stress requires further investigation.

### 2.9. Preliminary Functional Analysis of StGST7 in Yeast Under Salt and Drought Stress

**Figure 10:**
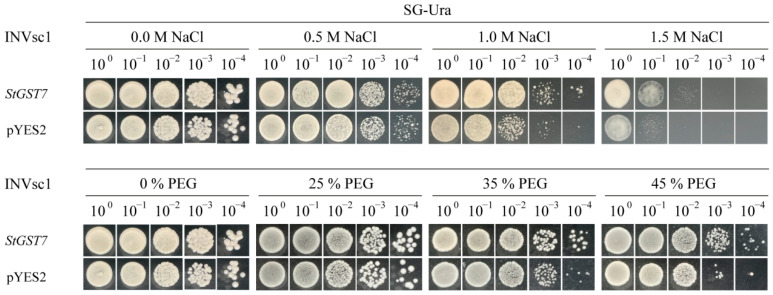
Functional validation of *StGST7* for stress tolerance in a yeast heterologous expression system. The transgenic yeast strains overexpressing the *StGST7* gene and the corresponding empty vector (EV) controls were adjusted to an identical initial cell density and subjected to serial dilutions (e.g., 10 to 10^−4^). Equal volumes of the diluted yeast cultures were then spotted onto normal media (control) and media supplemented with specified concentration gradients of NaCl (0.5, 1.0, 1.5 M for salt stress) and PEG (25, 35, 45% for simulated drought stress). The growth phenotypes and survival differences in the yeast colonies were observed and recorded after incubation at the appropriate temperature.

To ascertain the potential function of candidate gene *StGST7* in responding to salt and drought stress, this study obtained the coding sequence (695 bp) of this gene from cDNA clones of potato cultivar ‘Qingshu No. 9’ and constructed it into the yeast expression vector pYES2. The recombinant plasmid pYES2-StGST7 and the empty vector control pYES2-EV were separately transformed into the yeast strain INVSc1. Subsequently, the cells were treated for 5 days in media containing varying concentrations of sodium chloride (NaCl) and polyethylene glycol (PEG) to assess their tolerance to abiotic stress (Figure 10).

Under normal growth conditions, the growth status of the *StGST7* yeast heterologous expression strain was not significantly different from that of the control strain, indicating that the heterologous expression of this gene did not have a negative impact on the basic metabolism of yeast. However, under 0.5 M and 1.0 M NaCl stress, the *StGST7* gene significantly enhanced the growth vitality of yeast cells. Even under the extreme salt stress of 1.5 M NaCl, the *StGST7* yeast heterologous expression strain could still maintain growth at 10^−2^ and 10^−3^ dilution gradients, while the control strain could not survive at all. These findings demonstrate that the *StGST7* gene significantly enhances yeast salt tolerance.

Under PEG-simulated drought stress conditions, no significant growth differences were observed between the *StGST7* heterologous expression yeast strain and the control strain at a PEG concentration of 25%. However, as the stress concentration increased to 35% PEG, the heterologous expression strain exhibited a marked growth advantage. When the PEG concentration further increased to 45%, the *StGST7* yeast heterologous expression strain could still maintain growth at 10^−3^ and 10^−4^ dilution gradients, while the control strain almost completely lost its growth ability. Collectively, these results indicate that *StGST7* expression is associated with enhanced tolerance to salt and drought stress in yeast cells. These findings provide preliminary evidence that *StGST7* may be involved in abiotic stress responses, although its specific role in potato requires further validation.

## 3. Discussion

Plant genome sequences provide invaluable insights into the evolutionary significance of genes or gene families [42]. Gene family analysis based on a single reference genome serves as a crucial tool for deciphering gene evolution and function, encompassing core aspects such as family member identification, gene structure, phylogeny, and expression patterns [43]. However, this approach struggles to comprehensively reveal the presence/absence variation in genes prevalent within species, thereby limiting our understanding of the complete diversity and evolutionary dynamics of gene families. The pan-genome, by integrating the genomic information of multiple individuals, can systematically characterize the entire genetic diversity of a species. The identification of core and variable genes within the pan-genome provides a highly comprehensive perspective for investigating the evolution and functional divergence of this gene family [44]. Relevant studies indicate that genes associated with responses to biotic and abiotic stresses are frequently enriched among variable genes, suggesting the significant role of PAVs in adaptive evolution [45]. Consequently, gene family analysis employing a pan-genome strategy not only enables more accurate identification of family members and elucidation of their distribution patterns, but also provides deeper insights into the evolutionary mechanisms and functional potential of gene families in species differentiation, environmental adaptation, and breeding improvement. This approach thus furnishes richer genetic resources and theoretical foundations for crop genetic enhancement.

Recent pan-genomic analyses of gene families such as barley *bHLH* have provided important methodological insights for comprehensively elucidating the composition, evolution, and function of gene families at the species level [41]. Consequently, conducting a pan-genomic study of the potato GST gene family is particularly necessary. This will not only provide a comprehensive gene map for deepening our understanding of the genetic diversity of this family within potatoes but also lay a crucial theoretical foundation for subsequent functional analysis and genetic improvement. Compared to traditional analyses based on a single reference genome, pan-genomic analysis can more comprehensively reveal gene presence/absence variation. Presence/absence variants (PAVs) constitute a vital reservoir of plant genetic diversity, playing a significant role in species adaptive evolution, gene resource mining, and molecular marker development [46]. For instance, variable genes derived from PAVs in *Amborellaceae* plants have been demonstrated to enhance resistance to abiotic stresses and environmental adaptability [47]. Pan-genome studies of maize and rice TPS families revealed that only partial members constituted core genes present across all genomes, with the remainder being variable genes [48,49]. Similarly, cucumber *WOX* family members exhibited significant variation in numbers across different genomes [50]. These cases collectively demonstrate that PAV represents a universal mechanism for the quantitative and functional diversification of gene families across species and varieties. Based on the identification of 2940 members of the glutathione S-transferase (GST) gene family across 45 diploid potato varieties, this study systematically identified 1695 GST family members (present in at least two varieties) and classified them into eight subfamilies. Compared to the reference genome DM (containing only 46 members), pan-genomic analysis identified an additional 1649 members, fully demonstrating its comprehensive nature. Significant variation in the number of *GST* genes was observed among different potato germplasm lines, with PG6059 containing the highest number of members (104) and PG5018 the lowest (37). This widespread PAV phenomenon indicates that during domestication and evolution, certain *GST* genes were lost or acquired in distinct potato lineages, potentially forming a crucial genetic basis for trait differences among varieties. It is noteworthy that, based on a gene family analysis, Shi et al. [15] classified glutathione S-transferase (GST) family members in tetraploid potato (C88) into ten subgroups: Phi, TCHQD, Theta, Omega, Zeta, MAPEG, DHAR, EF1Bγ, Lambda, and Tau. By contrast, our phylogenetic analysis of *StGST* genes from 45 diploid potato accessions revealed that none of the 1695 identified *StGST* genes were assigned to the Theta, TCHQD, DHAR, or MAPEG subgroups. This discrepancy may be attributable to differences in ploidy level, sequence annotation quality, gene prediction completeness, identification strategies, or phylogenetic classification criteria. Thus, the apparent absence of these subgroups should be interpreted with caution rather than taken as definitive evidence of their biological absence in diploid potato. Further comparative genomic analyses and functional validation are needed to clarify the classification and evolutionary history of these GST subgroups in potato. Structural variations (SVs), encompassing inversions, duplications, deletions, and translocations, constitute the fundamental genetic basis driving plant trait diversification, environmental adaptation, and species evolution [51]. For instance, in peach, a 1.67 Mb heterozygous inversion directly conferred the formation of flattened fruit morphology [52]; tomato pan-genome studies further reveal SVs’ pivotal role in regulating gene expression, shaping phenotypic variation between accession, and enhancing stress resistance [53]. Our pan-genomic analysis of the potato GST gene family confirms that SVs are equally significant factors causing extensive variation in the number of family members, their genomic distribution, and gene structure. Compared to a single reference genome, multiple potato germplasm resources exhibit SV-mediated presence/absence variation and copy number differences in *GST* genes. This may directly determine the functional potential of different germplasm in stress response and metabolic regulation. The mechanisms by which SVs influence gene function are complex and diverse, extending beyond mere gene presence/absence. They frequently act by altering gene regulatory and coding sequences: SVs in promoter regions (e.g., insertions/deletions) may directly regulate transcriptional activity. For instance, specific promoter variants in maize can upregulate *bZIP68* expression, reducing cold tolerance [54], or enhance *ZmWAKL* expression to improve disease resistance [55]. Analysis of the potato *StGST* gene family in this study revealed that despite numerous SVs in coding and flanking regions, their overall impact on gene expression was limited. Only *StGST42* expression showed significant correlation with SVs, suggesting this family may possess high transcriptional stability during evolution. This finding aligns with observations in the ARF family, indicating that widespread SVs do not necessarily induce significant rearrangement of expression profiles [56]. However, analysis of conserved motifs revealed that while core domains (motifs 1–4, 7) remained fully conserved, certain auxiliary motifs were absent in specific cultivars. Concurrently, structural variations were observed in the 3′ untranslated regions (UTRs) across multiple accession, such as a significant increase in the number of units in this region for PG6055 and PG6148. This indicates that SVs may still indirectly regulate protein function or mRNA metabolic stability by altering protein domain composition or influencing post-transcriptional regulatory processes. This mechanism aligns with findings in maize and rice TPS families, where structural variation promotes the generation of atypical genes [48,49]. In summary, the potato GST gene family may buffer the impact of SVs by maintaining core structural conservation and transcriptional homeostasis, with variation primarily manifesting at the level of structural plasticity. For instance, coding region variations may generate functionally distinct subtypes (similar to the truncated variant of maize *ZmNLP8*), while UTR variations may influence mRNA stability and translation efficiency. Future studies should integrate multi-omics and functional experiments to systematically elucidate the specific regulatory networks of SVs in potato stress responses.

Extensive research indicates that GSTs play a pivotal role in plant responses to abiotic stress. In species such as *Arabidopsis*, rice, and tomato, overexpression of certain *GST* genes (e.g., *OsGSTU4*, *LeGSTU2*, *MruGSTU39*) significantly enhances plant drought tolerance, primarily through mechanisms involving enhanced antioxidant defense and reduced reactive oxygen species accumulation [5,57,58]. Concurrently, epigenetic regulation—particularly DNA methylation/demethylation dynamics—has been demonstrated to modulate stress-responsive gene expression. For instance, salt stress in maize induces demethylation of the *ZmGST* gene promoter region, thereby upregulating its expression [59]. Shi et al. [60] found that some *StGST* genes may be regulated by DNA methylation pathways during drought response in potato. In our study, *StGST7* clustered in the same clade as *AtGSTF11/F12* from *Arabidopsis* and was upregulated under salt and high-temperature stresses. It may participate in potato stress responses by regulating secondary metabolite biosynthesis and SA signaling pathways. In this study, we analyzed the expression patterns of *StGSTs* in the DM under salt, drought, and high-temperature stresses, and found that 29, 16, and 28 *StGSTs* were differentially expressed (|log_2_FC| > 1), respectively. Among them, seven *StGSTs* were differentially expressed under all three stress conditions. By further integrating RNA-seq data from tetraploid potato at different time points under salt stress, we identified candidate genes—*StGST129*, *StGST74*, *StGST138*, and *StGST7*—that may be involved in potato responses to abiotic stress. Interestingly, *StGST129* and *StGST74* are both orthologous to *AtGSTU8* (*AT3G09270*). Previous studies found that *AtGSTU8* responds to osmotic and oxidative stress signals and functions in glutathione-dependent detoxification and antioxidant defense [61,62]. Therefore, we speculated that *StGST129* and *StGST74* may have functions similar to those of *AtGSTU8* and participate in potato stress responses. Although *StGST138* was not an ortholog of *AtGSTU8*, the Blastp results revealed that it shares the highest sequence similarity with *AtGSTU8* in *Arabidopsis*, hypothesizing that *StGST138* may also share analogous functions with *AtGSTU8.* Furthermore, *AtGSTF11/F12* (AT3G03190 and AT5G17220) have been reported to function as enzymes or ligand-binding proteins involved in secondary metabolite synthesis and SA signaling [63,64]. In our study, *StGST7* clustered with *AtGSTF11/F12* and was upregulated under salt and high-temperature stresses, suggesting that it may also participate in potato stress responses through pathways related to secondary metabolism and SA signaling. Comparative analysis between different cultivars revealed differences in the response timing and magnitude of certain genes in the ‘Atlantic’ and ‘Qingshu No. 9’ cultivars. This indicates that *StGST* genes may participate in potato responses to salt stress through divergent regulatory pathways. Notably, some stress-responsive genes exhibited distinct tissue-specific expression patterns. For example, *StGST11* and *StGST121*, which are highly expressed in tubers, were also significantly induced during the late stage of salt stress, suggesting that they possess dual functions in the stress adaptation of storage organs [65,66].

Co-expression network analysis supports a regulatory model in which multiple transcription factor (TF) families jointly control *StGST* genes to balance stress responsiveness with growth maintenance. In this model, *StGST7* may serve as a key node under bidirectional regulation, showing positive association with bHLH096 but negative association with EIN3 and SCL4, thereby integrating stress signaling with developmental homeostasis [67]. Meanwhile, distinct TF preferences among *StGST* members (e.g., *StGST19* with SCL14/GEBPL/TRB1 versus *StGST10* with YY1/HSL1/RTV1/HHO2) indicate functional specialization that increases regulatory flexibility under variable environments. Previous studies have shown that TF families such as SCL, bZIP, and bHLH likely act predominantly as positive regulators of stress responses, whereas SANT/MYB and EIN3 may provide negative feedback linked to growth regulation and stress attenuation [15]. The appearance of atypical components such as RTV1 further suggests that *StGST* regulation may extend beyond transcription to post-transcriptional processes (e.g., protein trafficking/localization), together forming a multi-layer network that optimizes resource allocation in the growth–defense trade-off [68]. Collectively, these findings offer novel insights and essential genetic resources for unraveling the multi-level regulatory networks and precise functions of potato GSTs during abiotic stress adaptation.

The yeast heterologous expression system provides an efficient platform for evaluating gene functions in responses to abiotic stresses [69]. This study employed this system to validate the potato *StGST7* gene, revealing that heterologous expression of *StGST7* significantly enhanced the tolerance of transgenic yeast to salt and drought stresses. This suggests the gene may participate in the regulatory network governing potato adaptation to environmental adversities. Glutathione S-transferase (GST), a key enzyme in plant responses to abiotic stress, primarily functions through participation in reactive oxygen species scavenging [70], detoxification metabolism, and maintenance of cellular redox balance [71]. Under salt stress, plant cells frequently face dual challenges of oxidative damage and ionic toxicity [72]. Upregulation of *GST* genes aids in clearing excess ROS accumulation and participates in detoxification processes of stress-induced secondary metabolites [71]. It can be speculated that *StGST7* may function in a similar manner in yeast cells, enhancing their tolerance to salt stress. During PEG-simulated drought stress, *StGST7* overexpression similarly enhanced yeast cell survival, yet this protective effect exhibited marked stress-intensity dependence: under mild drought stress (25% PEG), there was no significant difference in growth between the overexpression strain and the control strain; however, under moderate and severe drought stress (35% and 45% PEG), overexpression of *StGST7* significantly enhanced the growth vitality of yeast cells. This phenomenon suggests that *StGST7* may function as an “enhancer” rather than a fundamental regulator in stress responses, with its role becoming prominent when stress exceeds the tolerance threshold of endogenous defense systems. It also indicates that *StGST7*-mediated drought stress responses may depend on specific signal intensities or metabolic states. In summary, this study utilized a yeast heterologous expression system to confirm the positive role of *StGST7* in enhancing tolerance to salt and drought stress. This provides crucial insights for subsequent validation of its biological function in potato plants and for elucidating its association with reactive oxygen species scavenging, sulphur metabolism, and redox regulatory networks. Subsequent studies should integrate plant genetic transformation, physiological and biochemical analyses, and molecular interaction experiments to systematically elucidate the application potential of *StGST7* in crop stress tolerance improvement and its underlying regulatory mechanisms.

## 4. Materials and Methods

### 4.1. Identification of the GST Gene Family and Presence/Absence Variation Analysis

To identify members of the GST gene family in potato (*Solanum tuberosum*), a total of 2940 *GST* pangenome and corresponding annotation files of the potato pangenome were obtained from the Potato Pangenome Database (http://solomics.agis.org.cn/potato/, http://218.17.88.60/potato/ accessed on 1 December 2025). The potato reference genome (DM v6.1) and its gene annotation files were downloaded from Spud DB (http://spuddb.uga.edu/ accessed on 1 December 2025). To maintain clarity, consistency, and traceability in gene nomenclature, and to avoid introducing misleading annotations, we adopted a systematic and neutral naming strategy based on the order of gene identification. It should be noted that a total of 2940 *GST*-related gene IDs were identified from the pan-genome dataset in this study. Because some homologous genes, allelic variants, or corresponding genes from different accessions shared the same gene name, these gene IDs were ultimately grouped into 1695 non-redundant *StGST* nomenclature entries, which were named *StGST1*–*StGST1695*. Detailed information, including gene names, accession names, and corresponding gene IDs, is provided in Supplementary Table S1. Hidden Markov model (HMM) profiles of the *GST* conserved domains, including GST-N (PF02798) and GST-C (PF00043), were retrieved from the Pfam database (http://pfam.xfam.org). Candidate *GST* genes were identified by searching the pangenome protein sequences against the *GST* HMM profiles using the HMMER program (*E*-value *<* 10^−5^), followed by homology-based confirmation [73]. All preliminary *GST* candidates at the genome-wide level were identified and further submitted to the SMART database (http://smart.embl.de/ accessed on 9 December 2025) and the Conserved Domain Database (https://www.ncbi.nlm.nih.gov/cdd/ accessed on 9 December 2025) [74,75] with domain verification.

Presence/absence variation (PAV) of *GST* genes across the potato pangenome was analyzed using a strategy similar to that described by Huang et al. [76]. For each *GST* gene, presence or absence information across individual accessions was extracted from the pangenome dataset, and a binary PAV matrix was constructed. During *GST* gene identification, for loci with multiple transcript isoforms, only one representative transcript was retained for subsequent analysis, with preference given to the longest protein-coding transcript. Candidate *GST* genes were further screened based on the presence of conserved *GST* domains, and entries lacking key domains, containing incomplete coding sequences, or representing redundancy caused by alternative splicing were removed. For GST-related gene IDs from different accessions that were highly homologous or potentially represented allelic variants, we integrated sequence similarity, phylogenetic relationships, and accession information to organize and merge these entries, thereby reducing redundant counts. Following the analytical framework used by Zhao et al. [77] for the maize (*Zea mays*) GATA gene family, the PAV matrix was visualized using the TBtools II (v2.301) [75], and a heatmap was generated to illustrate the distribution patterns of *GST* genes across potato accessions.

### 4.2. Phylogenetic Analysis

To investigate the evolutionary relationships of *GST* genes, GST protein sequences from potato and *Arabidopsis thaliana* were used to construct a phylogenetic tree. *Arabidopsis* GST protein sequences were obtained from the TAIR10 database (https://www.arabidopsis.org/ accessed on 10 December 2025). Multiple sequence alignment was performed using MAFFT v7.490 [78], and a maximum likelihood phylogenetic tree was constructed using IQ-TREE v2.3.6 [79]. The resulting tree was visualized and annotated using iTOL v6 (https://itol.embl.de/ accessed on 10 December 2025) [80].

### 4.3. Ka/Ks Ratio Analysis

Protein sequences and corresponding coding sequences (CDSs) of potato *GST* genes were extracted to evaluate selective pressure during evolution. Homologous gene pairs were aligned using ParaAT v2.0 [81], and Ka, Ks, and Ka/Ks ratios were calculated using Ka/Ks Calculator v2 [82]. Genes with Ka/Ks ratios greater than 1 were considered to be under potential positive selection. Ridge plots and heatmaps were generated using R packages (v4.0.3) to visualize selection patterns among *GST* subclasses [83].

### 4.4. Structural Variation and Gene Expression Analysis

Structural variation (SV) data were obtained from the Potato Pangenome Database (http://solomics.agis.org.cn/potato/, http://218.17.88.60/potato/ accessed on 12 December 2025), and genotype information corresponding to *GST* genes was extracted from SV datasets (http://solomics.agis.org.cn/potato/ftp/variation/potato_VCF_44sp.vcf.gz accessed on 12 December 2025). Although Huang et al. [78] previously reported expression profiles of *GST* genes across different potato tissues, their dataset did not cover all accessions included in the pangenome. RNA-seq data from stolon tissues across multiple potato accessions were used to compare expression levels between *GST* genes affected by structural variation and those without nonsynonymous SVs. Statistical analysis was performed using the Wilcoxon rank-sum test to evaluate differences in expression among the examined indicators.

### 4.5. Analysis of Conserved Domains Affected by Structural Variation

GST protein sequences from accessions harboring extensive structural variation and from the reference genome were subjected to motif and conserved domain analyses. Conserved motifs were identified using MEME (http://meme-suite.org/meme/tools/meme accessed on 3 December 2025), and multiple sequence alignment was performed using MAFFT, focusing on GST-N(PF02798) and GST-C(PF00043) domains. Comparative analyses were conducted to assess the impact of structural variation on the integrity of conserved domains.

### 4.6. Synteny Analysis

To explore chromosomal distribution and duplication patterns of *GST* genes, synteny analysis was performed using BLASTp (v2.17.0) searches against the potato reference proteome (DM v6.1) (*E*-value < 1 × 10^−5^, m8 format), followed by collinearity detection using MCScanX (v1.0.0; GFF format). This analysis enabled the identification of duplicated gene pairs and provided insights into the evolutionary expansion of the *GST* gene family.

### 4.7. Plant Materials and Treatments

To investigate the dynamic response of the *StGST* gene under specific stress conditions and the variations between varieties, this study conducted salt stress experiments using tissue-cultured seedlings of the tetraploid potato varieties ‘Atlantic’ and ‘Qingshu No. 9’. The seedlings were precultured in a Murashige and Skoog (MS) liquid medium containing 3% (*w*/*v*) sucrose, adjusted to 6.0 ± 0.5. The culture conditions were 23 ± 1 °C, a 12-h light/12-h darkness cycle, and the cultivation lasted for 3 weeks. During the salt stress treatment, the seedlings were transferred to the MS liquid medium supplemented with 250 mM sodium chloride. Samples were collected at 0, 1, 3, 12, 24, and 72 h after the treatment. Each treatment had 3 biological replicates, with each replicate containing 3 bottles of medium and 5 seedlings per bottle. After sampling, the materials were rapidly frozen in liquid nitrogen and stored at −80 °C for RNA-seq and qPCR analysis. The RNA-seq sequencing was entrusted to Shanghai OE Biotech Co., Ltd. for execution.

### 4.8. RNA Extraction, qPCR, and Statistical Analysis

For quantitative real-time PCR (qPCR) analysis, total RNA from the collected samples was extracted using an RNA extraction kit (DP419, Tiangen, Beijing, China). RNA integrity was monitored via agarose gel electrophoresis, and its concentration was determined using a NanoDrop ND-2000 spectrophotometer (NanoDrop Technologies, Wilmington, DE, USA). A total of 2 µg of RNA per sample was used as the template for first-strand cDNA synthesis. The elimination of genomic DNA contamination and reverse transcription were carried out using the FastKing RT Kit with gDNase (KR116, Tiangen, Beijing, China). qPCR was performed on a CFX96 Touch™ Real-Time PCR Detection System (Bio-Rad, Hercules, CA, USA) using SuperReal PreMix Plus (SYBR Green FP205; Tiangen, Beijing, China) with three biological replicates. The thermocycling conditions were as follows: initial denaturation at 95 °C for 30 s, followed by 40 cycles of 95 °C for 5 s and 60 °C for 30 s, ending with a melting curve analysis from 65 °C to 95 °C. The amplification efficiency of each gene was evaluated using a standard curve generated from a cDNA gradient dilution. StEF-1α (AB061263) was selected as the internal reference gene. Relative gene expression levels were calculated using the 2^(−ΔΔCt)^ method. All gene-specific primers used for qPCR were synthesized by Sangon Biotech (Shanghai, China), and their sequences are listed in Table S5.

### 4.9. RNA-Seq Data Analysis

Total RNA from the aforementioned samples was used for RNA-seq library construction. Library preparation, including mRNA enrichment, RNA fragmentation, adapter ligation, size selection, PCR amplification, and high-throughput sequencing on the Illumina platform, was performed by OE Biotech Co., Ltd. (Shanghai, China). The raw sequencing data have been deposited in the NCBI Sequence Read Archive (SRA) under the BioProject accession number PRJNA1455211. To obtain high-quality clean reads, the raw sequencing data were subjected to quality control, including the removal of adapter sequences, reads containing ambiguous bases (N), and low-quality reads with Phred scores < 20. Subsequently, the clean reads were aligned to the potato reference genome PGSC_DM_v6.1 using Bowtie2 (v2.2.9) with default parameters. The reference genome was downloaded from the Solanaceae Genomics Resource at Michigan State University (http://solanaceae.plantbiology.msu.edu/pgsc_download.shtml accessed on 9 January 2026). Gene expression levels were quantified based on the alignment results. Differential expression analysis was performed using the edgeR package in R, and raw read counts were normalized using the trimmed mean of M-values (TMM) method. The statistical significance of differences in gene expression was assessed using the Wilcoxon rank-sum test, with a significance threshold of *p* < 0.008. Genes with |log_2_FC| ≥ 1 and a false discovery rate (FDR) < 0.05 were defined as significantly differentially expressed genes (DEGs). All analyses were performed using default parameters unless otherwise specified.

To study the expression patterns of *StGST* genes in various tissues (sepals, leaves, roots, stems, stolons, tubers, flowers, petioles, petals, stamens, pistils, and mature and immature fruits), under abiotic stress (salt treatment: 150 mM NaCl, 24 h; mannitol-induced drought stress: 260 µM mannitol, 24 h; heat treatment: 35 °C, 24 h), and under exogenous hormone treatments (50 µM ABA, 24 h; 10 µM IAA, 24 h; 50 µM GA_3_, 24 h; 10 µM BAP, 24 h), the same methods as mentioned above were used, Illumina RNA-seq data were downloaded from the PGSC (http://spuddb.uga.edu/), and TBtools software (v2.030) was used to generate the heatmap.

### 4.10. Co-Expression Networks Analysis

Based on the co-expression analysis, *GST* genes were designated as central nodes, and normalized transcriptome expression data were used to calculate the expression correlations between *GST* genes and other genes. Gene pairs with significant correlations were retained for constructing the co-expression network. Subsequently, module detection and hub gene analyses were performed to identify gene modules closely associated with *GST* genes, thereby providing insights into their potential functional associations and biological roles.

### 4.11. Cloning of Candidate Genes

The coding sequences (CDSs) of candidate gene *StGST7* was amplified using ‘Qingshu No. 9’ cDNA as a template. The primers employed for this amplification are detailed in Table S4, with the amplification products shown in Figure S1.

Gene amplification was performed using PrimeSTAR Max DNA Polymerase in a 25 µL reaction system. Subsequently, the pYES2 vector was linearised with EcoRI and BamHI enzymes, and the target gene was ultimately inserted into the vector via the In-Fusion Snap Assembly Cloning Kit (Clontech/Takara Bio USA, San Jose, CA, USA).

### 4.12. Yeast-Based Functional Analysis of StGST Under Salt and Drought Stress

The plasmid pYES2-*GST7* and the empty vector pYES2-EV were introduced into the yeast strain INVsc1 separately. The transformed products were spread on SD-Ura solid medium and cultured at 28 °C for 3 days. Single colonies were picked out, and positive clones were verified using colony PCR. In the stress tolerance experiment, the positively transformed yeast cells were inoculated into SG-Ura liquid medium and cultured at 28 °C with shaking for 16 h (OD ≈ 0.6). Subsequently, the cells were harvested and resuspended in sterile water, then diluted in a 10-fold gradient (1, 10^−1^, 10^−2^, 10^−3^, 10^−4^). A total of 5 µL of each dilution was spotted onto SG-Ura medium containing different concentrations of sodium chloride (0 M, 0.5 M, 1.0 M, 1.5 M) and polyethylene glycol PEG (0%, 25%, 35%, 45%). All plates were cultured at 28 °C for 5 days, after which the yeast growth phenotypes were observed and recorded.

## 5. Conclusions

In conclusion, this study provides the first systematic pan-genome-wide characterization of the glutathione S-transferase (GST) gene family in potato. It reveals substantial variation in the number of *StGST* genes across 45 accessions and identifies near-core, non-essential, and private genes. Further analyses indicated that structural variations at the pan-genome level can significantly affect the expression of some *StGST* genes. Expression profiling showed that *StGST* genes exhibit tissue-specific expression patterns, with some members predominantly expressed in tubers and others in leaves. Moreover, integrated analyses of co-expression networks and responses to abiotic stresses highlighted the important roles of *StGST* genes in potato responses to high-temperature, drought, and salt stress. In addition, *StGST7* was identified as a candidate stress-responsive gene, and preliminary functional validation using a yeast heterologous expression system suggested its potential role in osmotic and salt stress tolerance. In addition, this study provides important insights into the evolutionary patterns of the potato GST gene family and its roles in abiotic stress responses, offering a theoretical foundation for identifying stress-related candidate genes and for molecular breeding of stress-tolerant potato cultivars.

## Data Availability

Data are contained within the article and Supplementary Materials.

## Supplementary Materials

The following supporting information can be downloaded at: https://www.mdpi.com/article/10.3390/plants15101548/s1, Table S1. Nomenclature and Gene IDs of 2940 Potato Genes. Table S2. The RNA-seq data of *StGST* genes in different tissues. Table S3. The RNA-seq data of *StGST* genes under abiotic/biotic stresses and hormone treatments. Table S4. Expression changes in the *StGST* gene in sensitive varieties (Atlantic variety) and drought-tolerant varieties (Qingshu No. 9 variety) of potato under salt stress conditions. Table S5. The primer sequence. Figure S1. Sequence alignment of *StGST7* gene.
