# Supplementary material for: Pan-Genome-Wide Analysis and Expression Profiling of the Potato GST Gene Family
Source: Plants (Basel). 2026 May 19;15(10):1548. doi: 10.3390/plants15101548 (PMC13210584; doi:10.3390/plants15101548)
Supplement: Supplementary file 1 [file plants-15-01548-s001.zip › Figure S1.pdf]

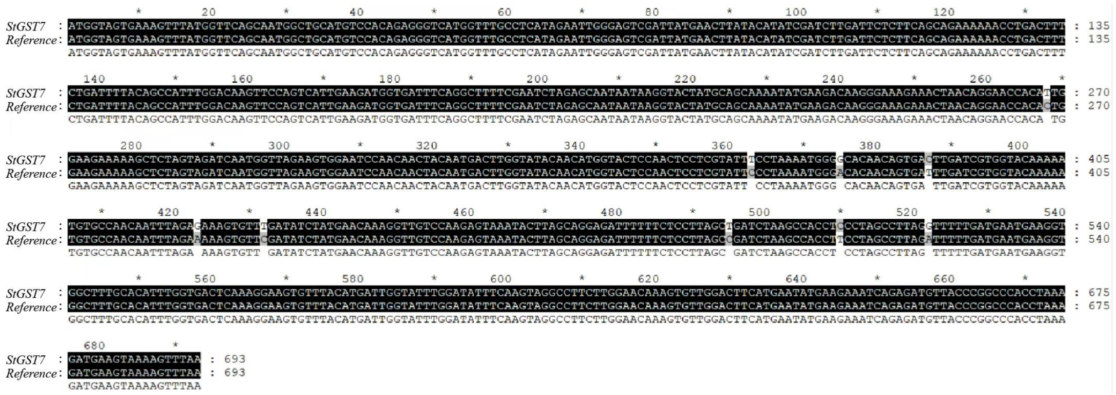

Figure S1. Sequence alignment of *StGST7* gene
